# Unfavourable Breeding Ground for Malaria

**Published:** 1918-08-24

**Authors:** 


### ANOPHELINE MOSQUITOES IN BRITAIN.

The map showing the known distribution in England and Wales of the anopheline mosquitoes, with explanatory text and notes by William Dickson Lang, M.A., printed by order of the Trustees of the British Museum, will be a revelation to most who study it, for few will remember that in times past malaria was a common disease in many parts of England, and few are aware that the varieties of mosquitoes known to carry the disease are found in most counties of Great Britain. But common they are and widely distributed. The closer the search the more are the mosquito-haunted localities. Attention has been called to the subject by the knowledge that many men who have fought in the Eastern campaigns are home again in England with the parasites in their blood. Fears are expressed lest these men should cause the disease again to show itself in endemic form. There is but small need for alarm. In all our recent history, every year hundreds and thousands of infected persons have been arriving in England; soldiers from India, West Africa, East Africa, Mauritius, the East and West India Islands, and civilians, British born or of foreign birth, from all these and many other infected places, have landed annually at our ports and scattered and settled through all the country-side, yet it is very seldom that a case of malaria originating in England is seen by any medical man even in the most mosquito-infested districts.

At first thought this is surprising. The explanation is that, although the infected persons and the mosquitoes that carry the disease to the healthy are constantly in the same neighbourhood; another important factor is absent, and that is mosquito-infested houses or dwelling-places. After the mosquito has become infected a period of several days elapses before the diseased mosquito is capable of infecting a human being. It must be remembered that malaria is probably as much a disease of the mosquito as of the man. The insect is not a mere vehicle of transport like the dirty-footed fly. There is a definite period of incubation and probably a period of active dis-ease in the literal meaning of the word, whilst the micro-organisms are working their way through the tissues of their host. The final escape of the germs from the salivary glands of the mosquito into the human host may be as great a relief to the mosquito as the coming of the sweating stage is to the man. If during the period of disease the mosquito is able to harbour in houses, it is ready at the right moment to inoculate the human beings that sleep in the rooms. The deeply shaded thatched and verandahed bungalows of India, the straw huts of Africa, the dirty houses and long accumulated rubbish of clothes in Macedonian villages, all shady, dark-cornered, little-dusted dwellings, are favourable to the concealment and secret lurking of diseased mosquitoes. It is not the thousands of fresh-hatched mosquitoes that swarm upon, irritate, and half devour him who lingers after sundown in the marshes and the jungles that infect a man, but the few that have fed on some infected British soldier, Indian servant, Greek labourer, or African porter, and remain by day in the thatch of the roof, in the hanging garments on a wall, or other place little subject to disturbance, and sally thence at nightfall. The infected mosquitoes that take their chance amidst innumerable foes in marshes, ponds, or sluggish rivers have but small chance to survive and convey the disease compared to the opportunity of the more domestic variety. In England the construction of the houses, the coldness of the nights, and the habits of the people all are against the domestication, the house haunting, of the mosquito, and, therefore, the probability of even a small increase of infection of English origin is small. All that the careful need do to make themselves reasonably safe is to see to it that any few mosquitoes that enter their houses are expelled or destroyed, and that no undisturbed corners are left to give them harbour. Even in hot, malarious countries it is probable that the making of scientifically constructed houses and a piped water supply would do more to lessen the incidence of malaria than the draining of many marshes.

